# Are Online Haters Psychopaths? Psychological Predictors of Online Hating Behavior

**DOI:** 10.3389/fpsyg.2020.00553

**Published:** 2020-03-27

**Authors:** Piotr Sorokowski, Marta Kowal, Przemysław Zdybek, Anna Oleszkiewicz

**Affiliations:** ^1^Institute of Psychology, University of Wrocław, Wrocław, Poland; ^2^University of Opole, Opole, Poland; ^3^Smell and Taste Clinic, Department of Otorhinolaryngology, Carl Gustav Carus Medical School, TU Dresden, Dresden, Germany

**Keywords:** online haters, online hating, hate speech, Psychopathy, Dark Triad

## Abstract

Despite growing prevalence of derogatory online behaviors, still little is known about psychological factors underlying this negative phenomenon. In the present study, we aimed to compare characteristics of persons who post hating and non-hating comments about Polish sports players during Winter Olympic Games in Pyeongchang (2018) on the Internet. Ninety-four Internet users (41% women) participated in the study, among which 46 posted hating comments. After 1 month, participants were invited to take part in a psychological survey, and filled the Dark Triad questionnaire, the Satisfaction with Life Scale, the Scale of Frustration, and the Scale of Envy. Results showed that high scores in Psychopathy subscale were significant predictors of posting hating comments online; high scores on the Envy Scale were marginally significant. Our findings provide initial evidence that persons who engage in derogatory online behavior have a high level of Psychopathy, but, contrary to previous studies, do not have elevated levels of other traits, commonly associated with disruptive behavior. Our research is one of the first to establish a psychological background of online haters, while setting a clear line between online hating and other derogatory online behaviors (e.g., trolling, cyber-bullying, or hatred speech).

## Introduction

Derogatory behavior has been long identified as a major social problem. It is not surprising that along with the growth of Internet popularity, such behaviors have been also observed in online settings (Blaya, 2019; Gauducheau, 2019; Johnson et al., 2019; Mathew et al., 2019), and thus identified as online hatred. Online hatred has been shown to inflate negative emotions (Lange, 2007), cause suicides (Marcus, 2018), and even lead to the assassination of public figures (e.g., Nyczka, 2019). Internet hate may affect not only human lives but also non-human targets. For instance, hate campaigns have been proven to be responsible for failures of big-budget movies (Bay, 2018). It seems that the phenomenon of online hate behavior is becoming more and more prevalent (Gagliardone, 2019), and so is the scholars attention to tackle this issue (Blaya, 2019; Derzsy, 2019; Johnson et al., 2019).

Despite the growing literature on hate behavior (Blaya, 2019), little is known about the personal characteristics of people who routinely engage in such behavior. There is not even an unanimous scholars’ agreement on what constitutes the definition of “Internet hating” or “haters,” as those terms have been referred to a broad range of derogatory behaviors (Shepherd et al., 2015; Cook et al., 2018). Primarily, it is worth to highlight the distinction between online hating and other forms of negative online activities. For instance, the purpose of hate speech is to express contempt and undermine the position of a given social group (according to e.g., race, gender, and nation) by expressing a disparaging opinion about that group, its particular members or its characteristic products (Nockleby, 2000). Thus, it is not considered hate speech to express a disparaging opinion about a person independently of their belonging to a given social group (Nockleby, 2000; Ortiz, 2019). Online hating, on the other hand, does not necessarily consists in expressing a disparaging opinion about a social group. It may be derogatory without in any way referring to the social position of a given person or object, and/or aiming at diminishing the social position of a group. Typical examples found on Facebook and other websites include comments that insult, for instance: public figures, sports person, actors (i.e., “*How can such a loser earn so much money!?*”; “*S/he must have got this job because s/he paid someone a lot or s/he has an “influential” uncle*”), deceased persons (i.e., “*This idiot drove so fast so s/he got what s/he deserved*”; “*What a stupid way to die, lol*”), or any other Internet users who post things online (i.e., active users of Facebook, YouTube, Instagram, Twitter, Twitch, and so on).

Scholars’ attention has been devoted to Internet trolls (Buckels et al., 2014; March, 2019), cyber-bullies (Fearn, 2017), and those who express hate speech (i.e., statements that are explicitly aimed at a certain social group) (Ortiz, 2019), with the lack of emphasis put on the recognition of personal characteristic of online haters (who may hate on a person regardless of the victims’ e.g., age, gender, or ethnic group). Each of those three derogatory online behaviors (i.e., trolling, cyber-bulling, and hate speech) have been connected with a slightly different psychological profile (Bishop, 2013, 2014). For instance, trolls have been told to score high on Psychopathy (while high scores on other Dark Tetrad characteristics have been inconsistently reported); and cyber-bullies have been told to score high only on sadism (for a review, see Moor and Anderson, 2019). Thus, it is reasonable to assume that those who routinely engage in online hating may exhibit certain, common characteristics, different on the type or severity from aforementioned behaviors. Nevertheless, to our knowledge, there is scarce data on the individual characteristics of online haters (who remain unidentified in previous research).

The main aim of the present study is to identify psychological predictors of posting hating comments online. Based on the initial literature review, we decided to focus on the following traits: Dark Triad (i.e., Narcissism, Psychopathy, and Machiavellianism), level of experienced frustration, level of experienced envy, and satisfaction with life. Dark Triad has been frequently used in previous studies on derogatory online behaviors (e.g., Golf-Papez and Veer, 2017; Sest and March, 2017; March, 2019; Moor and Anderson, 2019), and thus, examining Dark Triad traits should be the first step in establishing the commonalities and differences of online haters to other types of persons who exhibit negative behaviors online (e.g., trolls, cyber-bullies). A classic frustration-aggression hypothesis posits that frustration may lead to aggressive behaviors (Miller, 1941). More recently, Breuer and Elson (2017) overviewed numerous empirical research, and found evidence for the frustration-aggression link. Thus, it is reasonable to assume that if the frustration fosters aggressive behaviors, such occurrence may be even more pronounce in the online setup, as internet offers various ways to express verbal aggression (Wallace, 2015). Envy has been linked not only to one of the subscales of Dark Triad – Narcissism (Krizan and Johar, 2012), but also to indirect (verbal) aggression (Hofer and Busch, 2011), thus, we hypothesize that online haters may experience elevated levels of envy. Lastly, we expect that satisfaction with life could be negatively related to engaging in online hating, as being content with one’s life may buffer against both negative feelings, and aggressive behaviors (Valois et al., 2006). We will also examine the role of gender, as it was previously reported to be a predictor of negative online behaviors (Buckels et al., 2014; Craker and March, 2016; Sest and March, 2017).

## Methods

### Participants

Ninety-four Poles (41% women) aged 15–71 years (*M* = 33.4; SD = 13.9) participated in the study. Forty-six of them (further referred to as haters; 44% women, age *M* = 33.5; SD = 13.7) exhibited hating behavior (i.e., posted at least one comment, independently classified as online hating by two of the authors), and 48 persons (40% women, age *M* = 33.4; SD = 14.2) posted neutral comments (further referred to as non-haters; i.e., comments, independently classified as non-hating by two of the authors). Ethical approval of the study’s protocol was provided by the ethics committee at the Institute of Psychology (University of Wrocław).

### Procedure

Present study was conducted during Winter Olympic Games in Pyeongchang (2018). Authors searched for sports journals where performance of Polish Olympic Games contestants was reported and followed by comments through Facebook accounts. Online comments were independently screened and identified as hating or non-hating by two of the authors. Only if the agreement between authors was reached, the person who posted a given comment was identified either as an online hater or non-hater. We operationalized online hating posts as statements expressing a negative, insulting attitudes toward sports players; evaluative but not including constructive criticism. Exemplary hating comments included: “*She discredits our country and does it for taxpayers’ money, give me my money back!*,” “*Representing our country while being so ugly should be banned*.” Exemplary non-hating comments included positive statements: “*It’s alright, we keep our fingers crossed, next time s/he will do be better!*”; and negative statements: “*Considering the moderately good results throughout this season, during Olympic Game s/he performed rather badly. I think s/he wasn’t sufficiently prepared to this tournament*.” One month after closing ceremony of the Winter Olympic Games (2018), hating and non-hating persons received an invitation to participate in the psychological study via Facebook Messenger application.

### Measures

In the present study, we aimed to test, whether online haters differ from non-hating persons. To test Dark Triad, we used the Jonason and Webster (2010) questionnaire; polish adaptation by Czarna et al. (2016). The Dark Triad questionnaire had a high reliability for all subscales: Narcissism (Cronbach’s α = 0.89); Psychopathy (Cronbach’s α = 0.81); Machiavellianism (Cronbach’s α = 0.73). Moreover, we also used the Satisfaction with Life Scale (Diener et al., 1985; Polish adaptation by Jankowski, 2015). In our study, the Satisfaction with Life Scale had high reliability (Cronbach’s α = 0.85). As there are no established scales that would measure the trait of interest – experienced frustration, for the purpose of the present research, we decided to construct a short Scale of Frustration. This scale included two questions: “*I often experience unpleasant emotions, for instance: anger, anxiety, pain, as a result of not being able to fulfill one of my desires*”; “*I often experience unpleasant emotions, for instance: anger, anxiety, pain, as a result of not being able to achieve highly valued goals.*”; and participants responded to each item on a seven-point Likert scale (ranging from 1 – “*I definitely disagree*,” to 7 – “*I definitely agree*”). The two items were chosen based on the assumption that individuals experience frustration when they cannot fulfill their desires, or achieve their goals (Boyd, 1982; Crossman et al., 2009). The Scale of Frustration had a high reliability (Cronbach’s α = 0.88). The last scale, included in the present study, was the Scale of Envy, which was based on Tandoc et al. (2015) scale. We have used the three selected items: “*I do not think it is fair that some people have so much fun in their life, while others work really hard*”; “*Many people that do not deserve it, have a better life than me*”; “*Many people who do not deserve it, are happier than me*.” Participants responded to each item on a seven-point Likert scale (ranging from 1 – “*I definitely disagree*,” to 7 – “*I definitely agree*”). The Scale of Envy was highly reliable (Cronbach’s α = 0.89).

## Results

Table 1 shows means and standard deviations of both groups (i.e., haters and non-haters). A general overview of relationships between psychological variables, examined in the present study, is presented in Table 2. Only two variables significantly correlated with each other: persons that scored high on Machiavellianism subscale also expressed strong narcissistic tendencies; persons that expressed high frustration also scored high on Scale of Envy. In the next step, logistic regression was performed in order to investigate, which variables may account for posting hating online comments (see Table 3). Results showed that the strongest predictor of hating online comments was the Psychopathy subscale (β = 1.37, *Z* = 2.69, *p* < 0.001), whereas the Scale of Envy was close to reaching the statistical significance (β = 0.67, *Z* = 1.91, *p* = 0.056).

**TABLE 1:** Descriptive statistics for analyzed variables with regard to the group (i.e., haters, *N* = 46 and non-haters, *N* = 48).

	Haters		Non-haters	
	*M*	SD	*M*	SD
Age	33.47	13.65	33.27	14.18
Frustration	2.93	1.57	2.10	0.66
Envy	3.39	1.34	2.64	0.72
Narcissism	2.40	1.18	2.17	1.18
Psychopathy	2.11	1.07	1.56	0.49
Machiavellianism	2.18	0.90	2.04	0.82
Satisfaction with life	3.62	1.03	4.16	1.00

**TABLE 2:** Correlation matrix between psychological subscales for both groups (haters and non-haters) combined (Spearman’s rho).

	Frustration	Envy	Narcissism	Psychopathy	Machiavellianism	Satisfaction with life
Frustration	–					
Envy	0.32**	–				
Narcissism	0.15	0.19	–			
Psychopathy	0.09	–0.05	0.06	–		
Machiavellianism	0.16	0.06	0.67***	0.14	–	
Satisfaction with life	–0.15	–0.19	0.16	–0.01	0.01	–

**p < 0.01, ***p < 0.001.

**TABLE 3:** Binomial logistic regression on hating online comments.

	Hating online comments			
	β	*SE*	*Z*	*p*
Intercept	–3.70	2.21	–1.67	0.10
Age	0.00	0.02	0.23	0.82
Sex	–0.18	0.50	–0.36	0.72
Frustration	0.41	0.36	1.15	0.25
Envy	0.67	0.35	1.91	0.06
Narcissism	0.04	0.31	0.11	0.91
Psychopathy	1.37	0.51	2.69	0.01*
Machiavellianism	–0.08	0.38	–0.20	0.84
Satisfaction with life	–0.41	0.29	–1.42	0.16

Estimates represent the log odds of “hating online comments = 1” vs. “non-hating online comments = 0.” *p < 0.05.

## Discussion

In the present study, we sought to investigate whether certain psychological characteristics can predict posting hating comments online. Our results showed that high scores on the Psychopathy subscale was a significant predictor of posting hating comments online; whereas age, sex, high scores on Frustration, Envy, narcissism, Machiavellianism, and Satisfaction with Life scales were non-significant predictors. Interestingly, high scores on the Scale of Envy almost reached a statistical significance (on the level of a strong trend).

Our findings are in accord with previous studies, which provided evidence that negative online behaviors are associated with high levels of Psychopathy, in case of, for instance: trolls (Buckels et al., 2014; Golf-Papez and Veer, 2017; Sest and March, 2017; March, 2019; Moor and Anderson, 2019), cyber-bullies (Goodboy and Martin, 2015), and persons who exhibit hate speech (Withers et al., 2017). This result may not be surprising, as Psychopathy is characterized by impulsivity and thrill-seeking behavior (Paulhus and Williams, 2002), hence, high levels of impulsivity may foster impetuous behaviors, such as expressing a negative, insulting attitude/opinion toward someone or something, which is evaluative but, at the same time, does not include constructive criticism. One of the examples that portrays online hating behavior is the case of Polish Winter Olympic Games contestants, who have been widely attacked for their (unsatisfactory from the fans perspective) performance (Przegląd Sportowy, 2018), leading to negative reactions from the sports players – for instance, one of the players posted a provocative post on her Twitter page (i.e., “*You don’t know shit*”), which resulted in even more heated discussions on online hating, and its influence on mental well-being and performance of sports players (Kuczyñski, 2018).

Interestingly, posting online hating comments was not associated with higher levels of other Dark Triad traits (i.e., Narcissism and Machiavellianism), which were reported to correlate with personal characteristics of trolls (Buckels et al., 2014; March, 2019), cyber-bullies (Goodboy and Martin, 2015), and persons who post hate speech comments (Withers et al., 2017). Moreover, a high level of frustration and a low satisfaction of life has been previously linked to aggression (Valois et al., 2006; Breuer and Elson, 2017). Thus, we expected that both traits would be linked to verbal aggression (associated with online hating), but no such relationships were observed in the present study. Analysis revealed that there was only a weak, positive relationship between envy and hating comments, while, contrary to previous research, envy was not related to any of the subscales of Dark Triad (Krizan and Johar, 2012). Also, gender was not a significant predictor of online hating, which contradicts previous studies (Buckels et al., 2014; Craker and March, 2016; Sest and March, 2017).

One of the limitations of our study is that it reflects personal characteristics of only sports fans, and not the general population. At the same time, we believe that is also the strength of the methodology of our study, as sport is an important area of life, in which the vast majority of societies actively participate (Van Tuyckom and Scheerder, 2010; Bin and Lanjuan, 2019). Nevertheless, future studies should also focus on collecting data from people of various walks of life, scoping more general and broad topics discussed online. Moreover, the present research was conducted only among Polish Internet users. Despite the fact that Poles are said to be rather similar to other, European societies (Gross, 2004), future cross-cultural studies could provide some further evidence that the present findings may be generalized also to other societies. It would be also interesting to collect data from trolls, cyber-bullies, persons who engage in hate speech, and haters, as this could allow for explicit comparisons between persons who exhibit derogatory behaviors online.

Considering a reported increase in online hating (Blaya, 2019), predictions are that online hating behavior will become even more and more severe. Results of the present study are one of the first steps in broadening our understanding who the online haters are, which, in turn, may help identifying the best strategies for psychological interventions for haters, and creating counter-hating strategies.

## Data Availability Statement

The datasets generated for this study are available on request to the corresponding author.

## Ethics Statement

The studies involving human participants were reviewed and approved by Ethical Review Board of the Institute of Psychology at the University of Wrocław. Written informed consent for participation was not provided by the participants’ legal guardians/next of kin because: Ethical Committee of the Institute of Psychology (University of Wrocław) agreed that only the written consent of the participants (and not their legal guardian/next of kin) is required.

## Author Contributions

PS and AO: conception of the study. PS, MK, and PZ: data collection and the analyses. All authors contributed to drafting and reviewing the manuscript.

## Conflict of Interest

The authors declare that the research was conducted in the absence of any commercial or financial relationships that could be construed as a potential conflict of interest.
